# Site-specific chelation therapy with EDTA-loaded albumin nanoparticles reverses arterial calcification in a rat model of chronic kidney disease

**DOI:** 10.1038/s41598-019-39639-8

**Published:** 2019-02-22

**Authors:** Saketh R. Karamched, Nasim Nosoudi, Hannah E. Moreland, Aniqa Chowdhury, Naren R. Vyavahare

**Affiliations:** 1 Department of Bioengineering, Clemson University, Clemson, SC USA; 2 Department of Biomedical, Industrial and Human Factors Engineering, Wright State University, Dayton, OH USA

## Abstract

Medial arterial calcification (MAC) is a common outcome in diabetes and chronic kidney disease (CKD). It occurs as linear mineral deposits along the degraded elastin lamellae and is responsible for increased aortic stiffness and subsequent cardiovascular events. Current treatments for calcification, particularly in CKD, are predominantly focused on regulating the mineral disturbance and other risk factors. Ethylene diamine tetraacetic acid (EDTA), a chelating agent, can resorb mineral deposits, but the systemic delivery of EDTA may cause side effects such as hypocalcemia and bone resorption. We have developed elastin antibody conjugated albumin nanoparticles that target only degraded elastin in vasculature while sparing healthy tissues. In this study, we tested a targeted nanoparticle-based EDTA chelation therapy to reverse CKD-associated MAC. Renal failure was induced in Sprague-Dawley rats by a high adenine diet supplemented by high P and Ca for 28 days that led to MAC. Intravenous delivery of DiR dye-loaded nanoparticles confirmed targeting to vascular degraded elastin and calcification sites within 24 hours. Next, EDTA-loaded albumin nanoparticles conjugated with an anti-elastin antibody were intravenously injected twice a week for two weeks. The targeted nanoparticles delivered EDTA at the site of vascular calcification and reversed mineral deposits without any untoward effects. Systemic EDTA injections or blank nanoparticles were ineffective in reversing MAC. Reversal of calcification seems to be stable as it did not return after the treatment was stopped for an additional four weeks. Targeted EDTA chelation therapy successfully reversed calcification in this adenine rat model of CKD. We consider that targeted NP therapy will provide an attractive option to reverse calcification and has a high potential for clinical translation.

## Introduction

Patients with chronic kidney disease (CKD) have an elevated burden of cardiovascular disease (CVD) and compared to age-matched individuals with normal renal function^1,2^, are more likely to die due to CVD than to progress to renal failure. Although a cause of such excessive cardiovascular mortality has not been singled out, a major contributing factor is thought to be vascular calcification^1,3^.

Calcification in the arteries is of two types: In the intimal compartment of the arterial wall, it is associated with atherosclerotic disease and inflammation-causing stenosis; medial arterial calcification (MAC), also termed Monckeberg’s sclerosis, mostly occurs as linear deposits along the elastin lamellae in the media^4^. The latter is particularly prevalent and a common outcome of CKD, the result of CKD-factors specific to such as dysregulated mineral metabolism and secondary hyperparathyroidism^5^. MAC is an active biological process involving vascular smooth muscle cells (VSMCs) developing an osteoblast-like phenotype^6^. It leads to increased arterial stiffness, which in turn causes increased systolic blood pressure (SBP), pulse wave velocity (PWV), and pulse pressure (PP)^7,8^.

Current therapies to treat vascular calcification, particularly in CKD, predominantly consist of controlling the mineral disturbance and are mainly preventive in action^9,10^. Ethylene diamine tetraacetic acid (EDTA) is a promising chelating agent that can dissolve and remove calcium deposits if delivered near the calcification^11^. We demonstrated earlier that elastin antibody-conjugated nanoparticles (NPs) can be targeted to vascular calcification sites and that EDTA delivered by these NPs reverses elastin-specific MAC in a rat model of CaCl_2_ injury^12^. However, in that study, the aortic injury was created locally through a chemical insult, and systemic abnormalities usually associated with diseases like renal failure were not present.

Several research groups have employed the adenine-induced model of uremia and renal failure to characterize and investigate treatment methods for vascular calcification^13–15^. All these studies showed a common limitation that up to 50% of the rats fed with adenine diets do not show medial calcification in spite of a stable and comparable CKD. Price *et al*. introduced a variant to this model by lowering the amount of protein in the diet to 2.5% and higher amounts of P than in a standard rodent diet^16^. Renal pathology of this model mimics the clinical situation with the development of hyperphosphatemia, hypocalcemia, severe secondary hyperparathyroidism, and other biochemical changes such as elevated serum creatinine and elevated blood urea nitrogen that lead to vascular calcification. Hence, this model is considered a useful tool to study complications of arterial calcification in CKD patients and to evaluate therapeutic agents in the prevention of uremia-related MAC^17,18^. In the present study, we used the adenine diet with high P and high Ca-induced CKD rat model to test if albumin NPs loaded with EDTA delivered systemically would target calcific sites and remove vascular mineral deposition without the side effects associated with systemic EDTA chelation.

## Results

### Nanoparticle characterization

EDTA-loaded nanoparticles were prepared as described previously^12^. They were characterized by TEM and particle size analyzer for size and surface charge. NPs had a final average size of 254.42 ± 31.8 nm; Zeta potential of the NPs was measured as −24.42 ± 4.49 mv. The average yield of NPs after centrifugation was 53.18 ± 1.50%. EDTA loading into the NPs was 25.88 ± 0.172%. EDTA was released from these particles over a period of 72 hours. (Supplementary Fig. S1).

### Adenine-induced renal failure model

**Figure 1:**
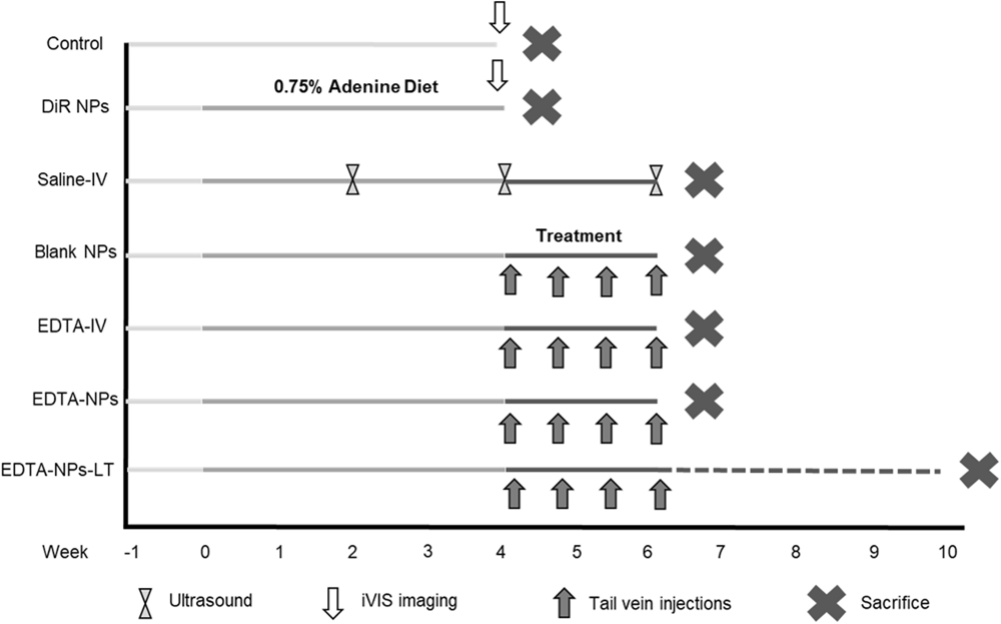
Schematic representation of the study design. The study consisted of seven groups of rats (n = 6 per group; 42 in total), two of which were used to study targeting elastin antibody conjugated NPs to the calcified aorta in the adenine model of chronic kidney disease. The remaining five groups were used to study different treatment regimens: Intravenous Saline (Saline-IV), anti-elastin antibody-conjugated blank NPs (Blank-NPs), Intravenous EDTA solution (EDTA-IV), anti-elastin antibody-conjugated NPs loaded with EDTA (EDTA-NPs) and anti-elastin antibody-conjugated NPs loaded with EDTA, in which the rats wereallowed to survive for 4 weeks after treatment (EDTA-NPs-LT). At the end of the experiment, animals were euthanized and blood and organs were harvested for analyses.

**Figure 2:**
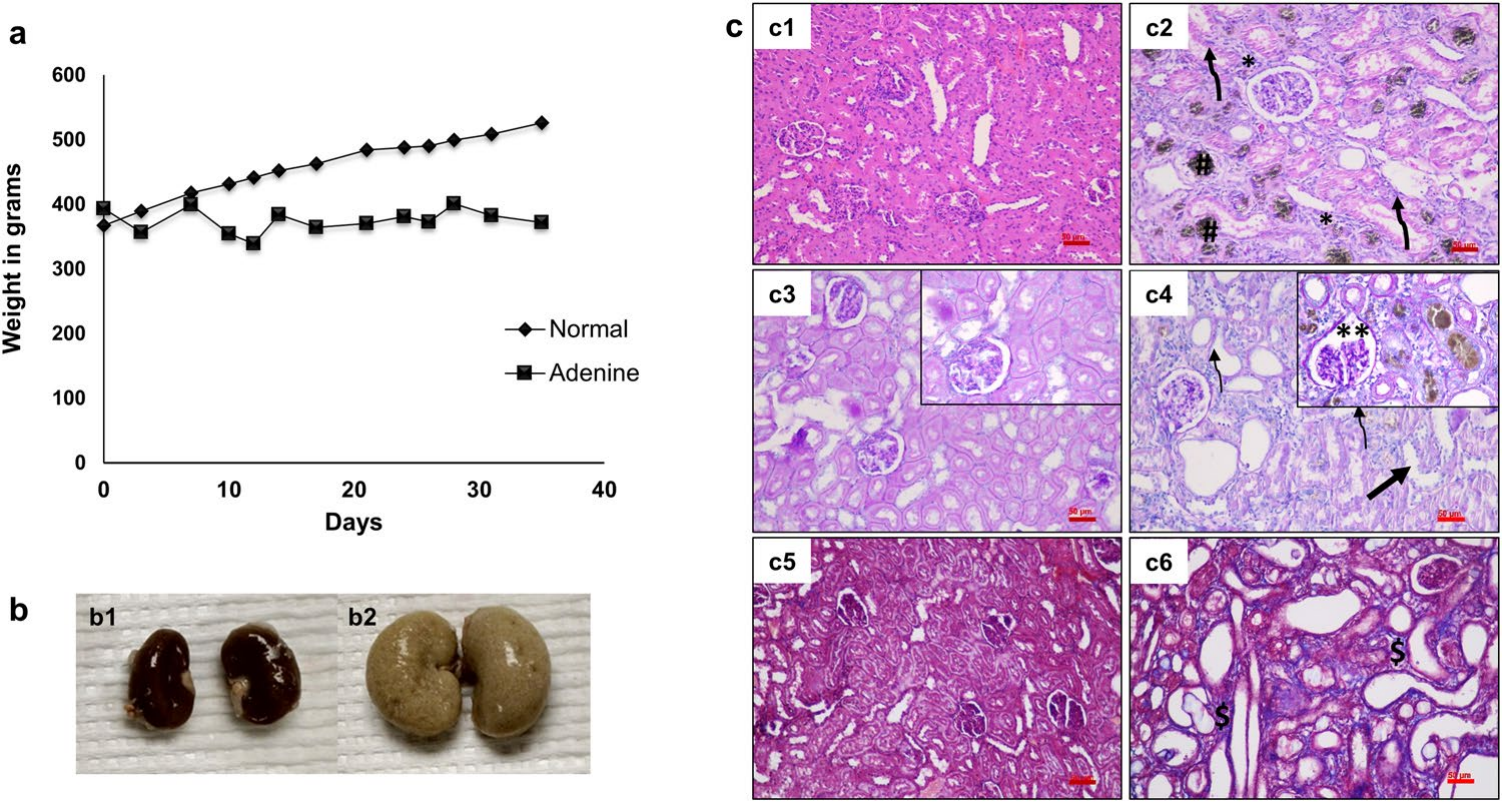
Body weights of rats, morphology and histology of kidneys, (n = 6 per group). (**a**) Body weights of the rats monitored during feeding normal or adenine diets. Adenine-fed rats lost weight compared to the rat-chow fed rats, but were returned to rat-chow diets intermittently to allow them to recover some of the lost weight and to ensure zero mortality. (**b**) Gross morphological images of standard diet fed (b1) and adenine diet fed (b2) rat kidneys; the latter show enlargement and extensive morphological damage. (**c**) Histological analyses of kidneys in the control (c1, c3, c5) and adenine-fed rat groups (c2, c4, c6). Representative changes on hematoxylin & eosin (H&E) staining (c1–c2), Periodic acid-Schiff reagent (PAS) staining (c3–c4), and Masson’s Trichrome (MT) stain (c5–c6). H&E stained sections from adenine-fed rats show infiltration of inflammatory cells (c2); tubular damage and crystal deposition of adenine are shown by arrows, * and ^#^ respectively. PAS stained sections showing intact brush borders of proximal tubules in control kidneys, but loss of brush border (thin arrows) and dilated tubules (thick arrow) in adenine-fed rats. Dilated bowman’s spaces are observed in the insets of PAS stained section of adenine diet-fed rats (**). Masson’s Trichrome images showed interstitial fibrosis (blue color $) indicating structural damage and remodeling in the adenine fed rats when compared to a control diet fed rats. *Scale bar 50 μm*.

Experimental flow chart for the experiments is shown in Fig. 1. Rats were fed adenine diets containing higher phosphate and calcium for 28 days to induce renal failure and vascular calcification. Body weights measured during the experiment indicated that the adenine diet-fed rats lost weight, possibly due to malnutrition and starvation (Fig. 2a). Modulating the adenine diet feeding as explained in the methods section enabled us to eliminate possible mortality in this model. All the animals survived until the end of the study, at which time they were electively euthanized.

Rats were randomly divided into seven groups of n = 6 each. Two of the seven groups were used to study targeting of a fluorescent dye, DiR-loaded NPs, to the calcified aortas while the remaining five groups were used for treatment studies with EDTA-loaded NPs. The entire study design is illustrated in Fig. 1 as well as described in more detail in the Methods section. Harvested kidneys from the adenine diet-fed rats showed extensive morphological damage from crystal deposition in comparison to a standard diet-fed kidney (Fig. 2b). Histology of kidneys from adenine diet-fed rats (Fig. 2C) revealed an increased infiltration of inflammatory cells and fibrosis. Structural changes were severe and predominant in these rats validated by increased vacuolation of tubules, tubular atrophy, an increased number of tubules with cell debris and dilated bowman’s spaces. Induction of CKD was further evidenced by higher serum creatinine and blood urea nitrogen (BUN) concentrations in the adenine diet-fed rats compared to normal animals. Furthermore, these animals also exhibited high serum phosphate and uric acid concentrations (Table 1).

**Table 1:** Serum biochemistry after induction of renal failure and following chelation therapy.

	Normal diet (n = 6)	Adenine diet for 4 wk (n = 6)	After therapy for two weeks (adenine diet was removed)				
			Saline-IV (n = 6)	Blank-NPs (n = 6)	EDTA-IV (n = 6)	EDTA-NPs (n = 6)	EDTA-NPs-LT (n = 6)
Serum Creatinine	0.25 ± 0.11	2.79 ± 1.30^*^	0.53 ± 0.10	0.70 ± 0.23	0.63 ± 1.1	0.73 ± 0.26	0.46 ± 0.00
Serum Phosphate	7.58 ± 0.25	11.42 ± 4.3	8.46 ± 0.54	8.66 ± 0.46	8.62 ± 0.48	7.95 ± 0.88	8.10 ± 0.50
Serum Calcium	10.00 ± 0.29	10.27 ± 0.42	10.167 ± 0.24	10.333 ± 0.359	10.15 ± 0.3	10.30 ± 0.30	10.36 ± 0.23
BUN	15.50 ± 1.50	53.34 ± 10.09	30.167 ± 3.1	31.66 ± 7.63	36.50 ± 5.85	46.16 ± 14.53	25.80 ± 5.11
Uric Acid	0.73 ± 0.12	1.44 ± 0.15	1.62 ± 0.18	1.58 ± 0.34	1.42 ± 0.21	1.317 ± 0.2	1.26 ± 0.1

^*^Represents statistical significance (p-value ≤ 0.05).

### Organ distribution of nanoparticles and targeting to the damaged aorta

**Figure 3:**
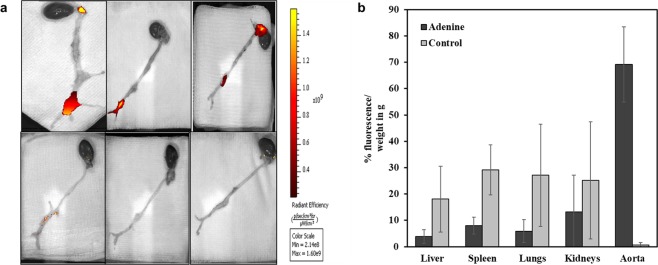
Nanoparticle targeting to diseased aortas, (n = 6 per group). (**a**) Nanoparticle accumulation in aorta following systemic intravenous injection of EL-DiR-NP twenty-eight days after adenine feeding. DIR NPs targeted the diseased regions twenty-four hours after delivery in 4 of 6 adenine-fed rats while the healthy regions were spared (top panel). Control diet-fed rat aortas do not show any targeting or accumulation of DIR NPs (lower panel). (**b**) Organ distribution of nanoparticles as measured by fluorescence intensity as imaged on IVIS imaging system twenty-four hours after injection. Nanoparticles are targeted to the aortas in adenine-fed rats while aortas of control diet rats show no targeting to the aorta.

**Figure 4:**
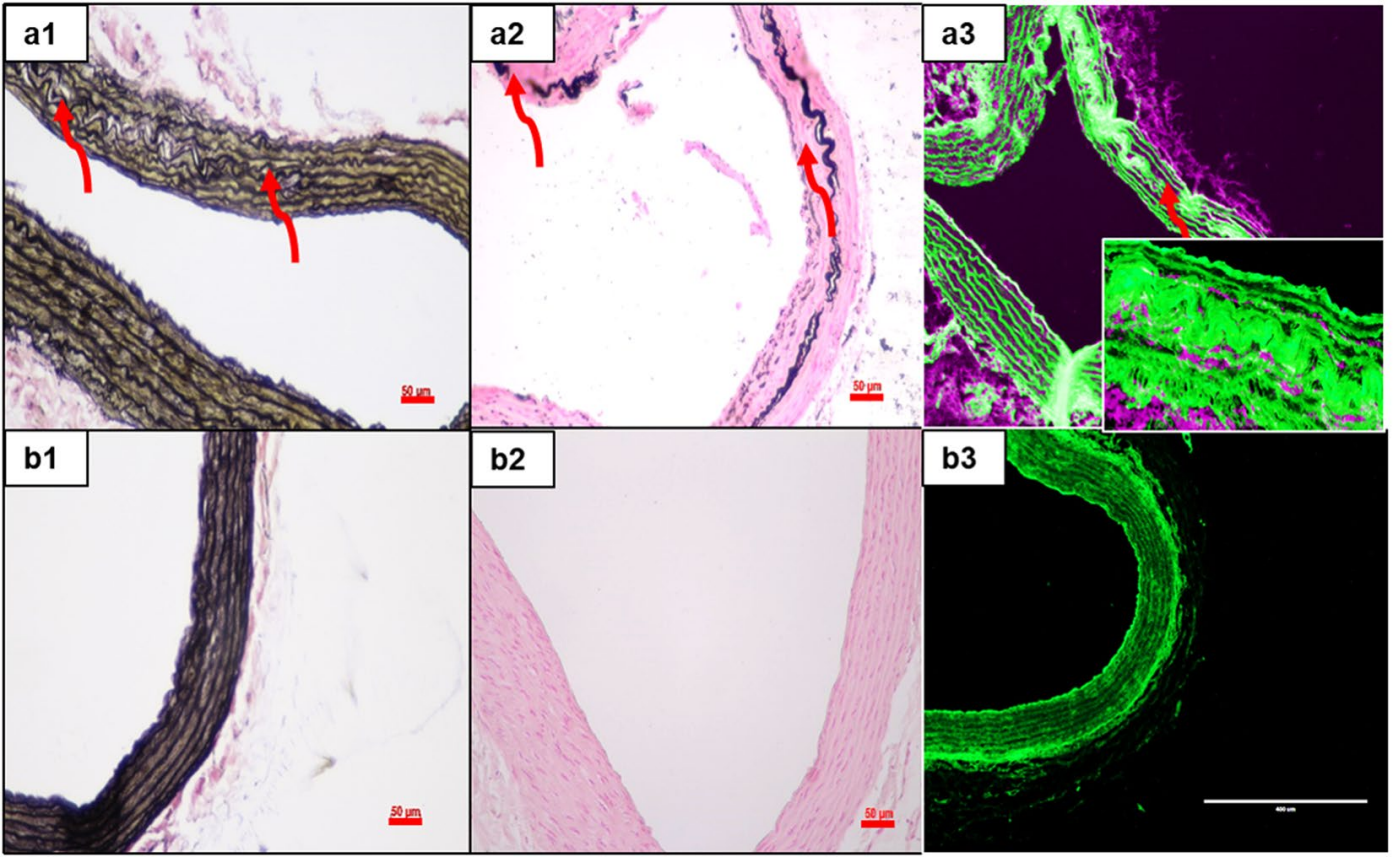
Histology of the aortas in control and adenine-fed rats, (n = 6 per group). Representative histological images of the aorta (panel a- Adenine diet and panel b- Normal diet): Verhoeff van Geison staining (**a1**,**b1**), Von Kossa staining (**a2**,**b2**) and Cy7 fluorescence (**a3**,**b3**). Verhoeff van Geison stain (**a1**) shows damage to the elastin fibers in the medial layer. Calcium deposits are observed in von Kossa stain (black) (**a2**) confirming calcification around the degraded (damaged) elastin fiber in the media. Cy7 fluorescence for DIR along with green autofluorescence of elastin in the adenine rat (**a3**) indicates NPs targeted the adventitial side and accumulation close to the degraded elastin lamina (Inset). Sections from aortas of rats fed normal diet showed neither degradation nor aortic calcification or nanoparticle targeting (**b1**–**b3**). *Scale bar 50* *μm*.

Elastin antibody-conjugated and DiR dye-loaded NPs were injected through the rats’ tail vein after 28 days of feeding the adenine diet. The NPs targeted the calcified and degraded aortic elastic lamina sites within 24 hours while sparing the healthy regions of the aorta in 4 of 6 rats injected with DiR-NPs, which correlated with elastin damage. Animals that were fed normal diets did not show any targeting of NPs to the aorta (Fig. 3a). The percentage fluorescence intensity normalized to the tissue weight for various organs showed 69.16 ± 14.27% targeting of NPs to the calcification sites (Fig. 3b). Representative histological sections of the aortas showed that there was indeed degraded elastic lamina in the medial layers of the aorta as shown by Verhoeff-van Gieson (VVG) staining (Fig. 4,a1) and calcification of the damaged/degraded elastic lamina as shown by von Kossa staining (Fig. 4,a2). NPs loaded with DiR dye were infiltrating the calcification site through adventitia in the adenine diet-fed rats and reached the medial calcification sites (Fig. 4,a3).

### Targeted EDTA Chelation Therapy leads to reversal of calcification

**Figure 5:**
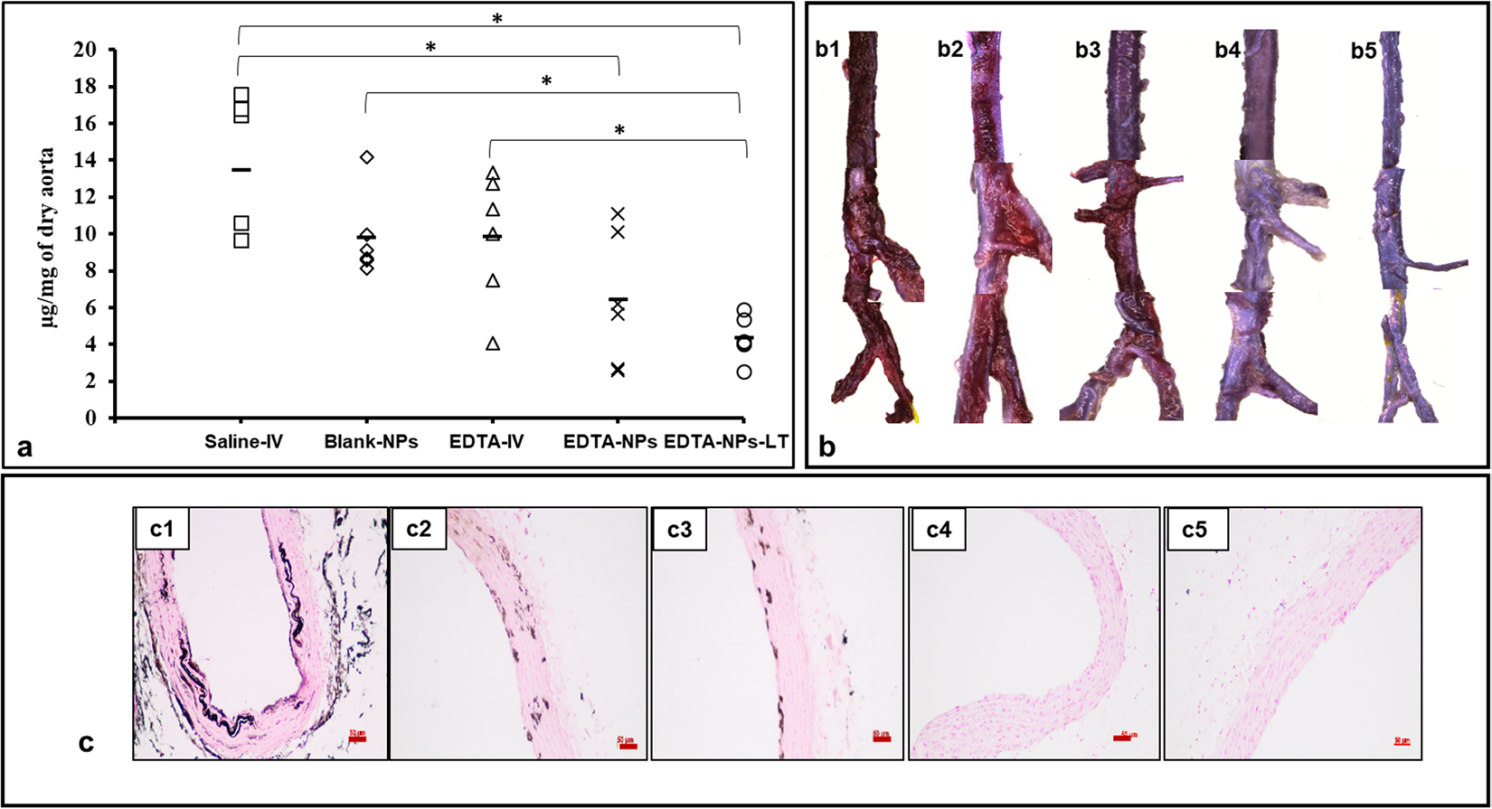
Reversal of aortic calcification by nanoparticle therapy, (n = 6 per group). (**a**) Calcium quantification in the aortas of all the treatment groups. Saline-IV group showed the maximum amount of calcium per aortic dry weight. Blank-NPs and EDTA-IV groups did not show significantly reduced calcium levels. Both EDTA-NPs and EDTA-NPs-LT groups show significant reduction in calcium compared to Saline-IV group. *(One-way ANOVA with Tukey’s HSD) represents statistical significance. Solid line represents mean value. (**b**) Whole mount aortas stained with alizarin red S to visualize calcium indicated that Saline-IV (b1) and Blank-NPs (b2) group have bright red staining for Ca while slightly reduced red staining in the EDTA-IV group (b3). Alizarin red stain is completely absent in EDTA-NPs (b4) and EDTA-NPs-LT groups (b5). (**c**) Paraffin-embedded sections of the aortas stained with von Kossa stain (black) revealed Ca deposits in Saline-IV (c1) and Blank-NPs groups (c2) along the degraded elastic lamina and these calcium deposits were slightly reduced in EDTA-IV group (c3). EDTA-NPs (c4) and EDTA-NPs-LT groups (c5) showed no calcium deposits. *Scale bar 50* *μm*.

Quantitative calcium content of the aortic tissues showed the highest calcium content (13.47 ± 3.5 µg/mg of the aorta) in intravenous saline (Saline-IV) group. There was a slight decrease in systemic intravenous EDTA (EDTA-IV) group (9.82 ± 3.3% µg/mg), but the decrease was not statistically significant. EDTA-loaded targeted NPs (EDTA-NPs) group showed the least calcium content (6.4 ± 3.2% µg/mg). This was significantly different from the Saline-IV group (Fig. 5a), which was further reduced in EDTA-NPs-LT group suggesting that removal of calcification continued even after NP therapy was halted. Aortic calcification evaluated by stereomicroscopic images of alizarin red-stained whole aortas (Fig. 5b) corroborated the quantitative calcium results. Rats treated with EDTA-NPs and EDTA-NPs-LT showed significant elimination of the calcific spots, suggesting reversal of mineral deposition. Representative histology of the aortas stained with von Kossa stain (Fig. 5c) showed pronounced calcification of the medial layer in the Saline-IV (c1) and Blank-NP (c2) groups. This was to some extent reduced in aortas treated with EDTA-IV (c3), but it was completely absent in EDTA NPs (c4) and EDTA-NPs-LT groups (c5), clearly suggesting that EDTA-removed calcium deposits and calcification did not return even after EDTA treatment was stopped for four weeks.

### Serum biochemistry following EDTA nanoparticle treatment

Serum biochemistry measurements following chelation therapy show reduced creatinine and P levels in all the groups, including the Saline-IV and Blank-NP groups (Table 1). This may be due to the adenine diet being discontinued during therapy. BUN, Uric Acid, however, remained high in all the groups. No morphological of histopathological differences were seen in kidneys from all the treatment groups compared to kidneys from adenine-fed rats before the treatment.

### Immunohistochemical analyses of the aorta for SMC phenotype and MMPs

**Figure 6:**
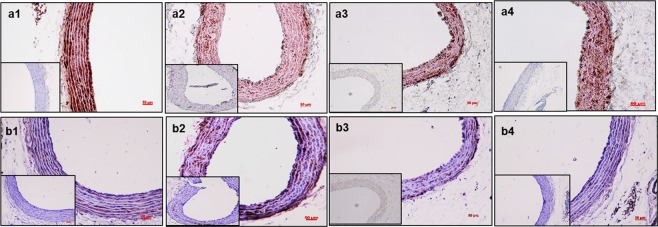
Immunohistochemical staining of the aortas to study VSMC status. (n = 6 per group). Representative images of IHC staining for anti-alpha smooth muscle actin for VSMC phenotype (**a1**–**a4**) and caspase-3 for apoptotic activity (**b1**–**b4**). Saline-IV group showed loss of VSMC phenotype (**a2**) compared to a normal diet rats (**a1**). Staining for anti-alpha smooth muscle actin is much more pronounced in both EDTA-NPs (**a3**) and EDTA-NPs-LT groups (**a4**). Caspase-3 staining revealed apoptotic cell death in Saline-IV group (**b2**) when compared with rats fed a normal diet fed (**b1**). Apoptotic activity is not as evident in either EDTA-NPs (**b3**) or EDTA-NPs-LT (**b4**) groups. Insets in each figure are negative controls without primary antibody. *Scale bar 50* *μm*.

Staining for alpha-smooth muscle actin indicated the loss of SMCs in the Saline-IV group due to calcification (Fig. 6,a2) compared to a normal diet-fed control rat (a1). SMC staining increased significantly in the EDTA-NPs and EDTA-NPs-LT groups (Fig. 6,a3,a4), indicating that the removal calcification allowed restoration of SMC phenotype in the medial layer. To explore if the loss of VSMCs was because of apoptosis, Caspase-3 staining with 3,3′-Diaminobenzidine chromogen was performed. Caspase-3 marker was not detectable in a normal diet-fed rat (Fig. 6,b1), and it increased around the calcific sites in Saline-IV group (Fig. 6,b2). Apoptotic activity was considerably reduced in EDTA NPs and EDTA-NPs-LT groups (Fig. 6,b3,b4), clearly suggesting that apoptotic cells were replaced by healthy cells.

**Figure 7:**
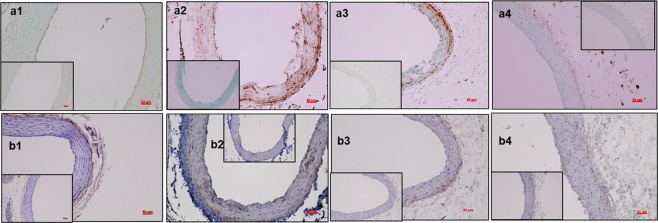
Immunohistochemical staining of the aortas to study MMP activity. (n = 6 per group). Representative images from IHC staining for matrix metalloproteinases MMP-2 (**a1**–**a4**) and MMP-9 (**b1**–**b4**). MMP-2 activity is easily identified in the calcified regions of media in Saline-IV group (**a2**), indicating that MMP-2 maybe involved in elastin breakdown and calcification, which was absent in a normal rat aorta (**a1**). MMP-2 levels were reduced after treatment with EDTA-NPs (**a3**) and almost absent in EDTA-NPs-LT group (**a4**). MMP-9 activity is present in the media in Saline-IV (**b2**) group and not in rats fed with a normal diet (**b1**). MMP-9 activity levels were not detected in either EDTA-NPs (**b3**) or EDTA-NPs-LT (**b4**) groups. Insets in each figure are negative controls without primary antibody. *Scale bar 50* *μm*.

Anti MMP-2 staining showed that MMP-2 activity was present in the calcified regions of the aorta in Saline-IV (Fig. 7,a2) with no detectable MMP activity in a normal diet fed rat (Fig. 7,a1). Treatment with EDTA-NPs (a3) slightly reduced MMP-2 activity, while EDTA-NPs-LT group showed the absence of MMP-2 staining in the medial layers (a4). Staining for MMP-9 also was present in the calcified regions of the medial layers of the adenine diet-fed rat aortas in Saline-IV (Fig. 7,b2) and absent in a normal rat aorta (Fig. 7,b1). MMP-9 activity was significantly reduced in both the EDTA NP treatment groups –EDTA-NPs (b3) and EDTA-NPs-LT (b4).

### *In vivo* ultrasound imaging

**Figure 8:**
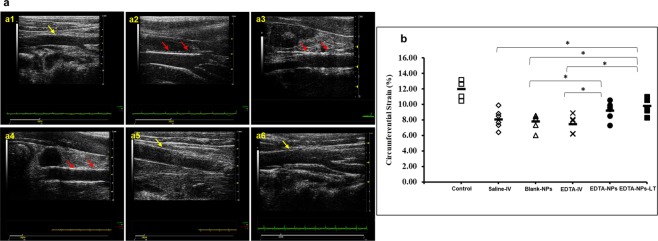
*In vivo* ultrasound imaging of the aortas, (n = 6 per group). (**a**) Representative 2D B-mode images from *in vivo* ultrasound scanning of aortas (a1–a6). Image from a healthy aorta (a1) does not indicate any sign of calcification. Aorta from Saline-IV group (a2) displayed bright and dense regions in the aortic wall, showing possible sites of calcium deposition (red arrows). Blank-NPs (a3) and EDTA-IV (a4)-treated aortas showed similar bright regions in the wall for calcification as indicated by red arrows, demonstrating that calcification reversal was not successful. EDTA-NPs (a5) or the EDTA-NPs-LT (a6) groups did not exhibit any such bright regions in the aorta, which confirmed reversal of calcification. (**b**) Circumferential strain, an aortic stiffness parameter, as calculated from the Green-LaGrange equation. Among the treatment groups, Saline-IV, Blank-NPs and EDTA-IV groups showed reduction in the strain values when compared to a healthy control rat, suggesting increased stiffness. Only EDTA-NPs and EDTA-NP-LT groups showed significantly improved strain values compared to other treatment groups although the improved values still did not reach healthy levels. *(One-way ANOVA with Tukey’s HSD) represents statistical significance. Solid line represents mean value.

Ultrasound images of abdominal aortas were obtained during adenine diet feeding and after therapy. Healthy aortas from standard chow-fed rats showed thin and elastic aortas (Fig. 8a,a1). In rats fed with the adenine diet, substantial calcification was seen in the medial layer of the abdominal aorta. Among the treatment groups, calcification was observed to be noticeably reduced only in the EDTA-NPs group; the Saline-IV, Blank-NPs and EDTA-IV groups all showed the persistence of calcification (Fig. 8a,a2,a5). Circumferential strains of the healthy aortas, as calculated from the Green-LaGrange strain equation, were 11.99 ± 1.043% (n = 6). Strains in the rats fed with adenine diet for 28 days showed decreased circumferential strains, suggesting stiffening of the artery due to aortic mineralization. Saline-IV, Blank-NPs and EDTA-IV groups did not reduce calcification; thus, aortic stiffness remained unimproved. Only EDTA-NPs group showed a statistically significant improvement in circumferential strain (9.22 ± 1.05%) compared to the rest of the groups, suggesting that removal of the mineral content led to more elastic aortas (Fig. 8b).

### Bone morphology and functional testing

Representative 2D-image from micro CT scans of the femurs about 10 mm below the trochanter revealed reduced mineralization in all the groups. EDTA-NPs group did not show any additional reduction in mineralization compared to any other group fed with adenine diet, indicating that EDTA NPs did not cause structural damage to the bone. (Supplementary Fig. S2a). Functional studies on bone stability indicated that the maximum load needed to fracture the femurs did not differ significantly among the four treatment groups. This confirmed that bone stability was not affected by EDTA-NPs treatment (Supplementary Fig. S2b).

## Discussion

Our goal was to test whether elastin-specific medial arterial calcification (MAC) could be reversed by targeted EDTA chelation therapy in a clinically relevant model of CKD. To our knowledge, we for the first time demonstrated that targeted nanoparticles injected systemically could target the calcified arteries and remove calcification in a CKD model in rats despite altered blood biochemistry.

MAC is highly prevalent in CKD, and traditional risk factors only such as hypertension and dyslipidemia do not completely account for the high calcification burden in the vasculature of the CKD dialysis population^3^. Calcification is enhanced by various risk factors common to CKD such as hyperphosphatemia, hypercalcemia, and high serum PTH. Hyperphosphatemia in particular, caused by reduced renal phosphate excretion and an increased calcium x phosphate product in the blood, is identified as a cause of accelerated medial calcification in CKD patients^19^. Over the last few years, several research groups have made use of the adenine model for studying mechanisms of MAC in CKD^14,16,17,20–22^. We were able to successfully induce CKD in rats fed with 0.75% adenine supplemented by high concentrations of Ca and P. Along with the expected structural damage to kidneys, biochemical abnormalities like elevated serum creatinine, serum phosphate, and higher levels of the BUN were seen. This closely resembled CRF in humans, making the model useful for the study of our targeted chelation therapy.

Several research groups have reported that one of the chief limitations of the adenine model is that a small dose of adenine (0.2%) causes inconsistent calcification, and a high dose (0.75%) causes excessive mortality^17,18^. In our studies, carefully controlled 0.75% adenine-feeding led to medial vascular calcification without undue mortality. Albumin NPs conjugated with elastin-antibody, which recognizes only damaged elastin in the vasculature, were used to deliver EDTA to the calcification sites in animals fed adenine diets. Fluorescence intensity of DiR dye-loaded animals showed accumulation of NPs at calcification sites. The healthy regions of the aorta in these animals and the entire aorta in the rats fed rat chow diets were spared, demonstrating that NPs targeted only degraded elastin. In previous studies, we showed that nanoparticles conjugated with generic IgG antibody do not target damaged elastin site^12,23–25^, suggesting that our elastin antibody conjugation led to site specificity. NPs also accumulated in liver and spleen, possibly because they are taken up by Kupffer cells in the liver and due to reticuloendothelial clearance^26^. This is very common for many nanoparticle therapies^27,28^. Moreover, we observed that the NPs entered the media from the adventitial side through the vasa vasorum^23,24^. Such adventitial delivery is advantageous: Often, intraluminal thrombus or atherosclerosis can hinder particles targeted from the lumen side.

Having established targeting efficiency of NPs to the calcified sites, we tested reversal of calcification by delivering EDTA-loaded NPs. We prepared albumin nanoparticles loaded with EDTA following an established procedure^12^. Previously, we showed that such nanoparticles release EDTA slowly over a period of 72 hours. EDTA is a strong chelator of metal ions and has been used as a treatment for metal poisoning. EDTA has also been considered as a possible treatment for coronary artery disease. From 2008–2013, a US clinical trial to assess chelation therapy (TACT) showed that an intravenous regimen with EDTA could modestly reduce the risk of adverse cardiovascular outcomes, but the results were not statistically significancant^29^. The study was not designed to show whether coronary calcification was removed, but only to assess the risk of cardiovascular outcomes. Because this study found a striking reduction of recurrent cardiovascular events in post-MI diabetic patients receiving EDTA disodium-based chelation therapy, the NIH recently initiated another clinical study (TACT 2) exclusively in diabetic patients^30^. Our previous studies also showed, both *in vitro and in vivo*, that when EDTA was delivered in proximity to calcific deposits, EDTA removed the calcification^11,12^. In this study, we chose to inject NPs twice a week for two weeks based on the release profile for EDTA: It was released from the NPs over a period of 72 hours. We included systemic EDTA injections as controls, at a dosage similar to the chelating agent released from our nanoparticles. After the two weeks of injections, we observed that the EDTA-loaded NPs reversed calcium deposits in the rat arteries, as confirmed by *in vivo* ultrasound, whole mount aorta alizarin red stain, histology with von Kossa stain, and quantitative calcium levels. When EDTA alone was injected systemically, calcification was reversed only slightly; therefore, multiple injections or a higher concentration of EDTA will be required to achieve reversal. That can lead to systemic side effects as shown previously with systemic EDTA therapy^13,31,32^. As expected, when the rats were injected with saline only and blank NPs as controls, the calcium deposits were not removed, and aortas remained calcified and stiff. We checked the status of kidneys in all animals at the end of the therapy by measuring blood biochemistry (Table 1). As the adenine diet was not continued during the two-week therapy, we did find reduced creatinine and P levels in all groups (including Saline-IV and Blank-NP groups) while BUN, uric acid remained high. This showed that EDTA therapy did not affect kidney function. Stopping the adenine diet might have improved kidney function in all groups, but once vascular calcification developed, it did not regress as can be seen in saline and Blank-NP groups. We saw a significant reduction in vascular calcification only after targeted therapy.

Elevated MMPs have been reported to have a strong correlation with elastic fiber degradation, stiffness, and calcification. Particularly, MMP-2 and MMP-9 up-regulation have been associated with arterial stiffening in diabetic patients with CKD^33^. Immunohistochemical staining for MMP-2 and MMP-9 showed that both were indeed present in the calcified regions of the aorta in adenine-fed rats. This suggests that MMP-2 and MMP-9 may be involved in elastin degradation and eventually causing elastin calcification in the adenine model. We showed that MMP-9 and MMP-9 knockout mice did not show vascular calcification in a CaCl_2_ injury model^34^. Significant reduction in both MMP-2 and MMP-9 after removal of calcification suggests that the inflammatory cascade is reversed after removal of calcification. It may be that locally delivered EDTA chelated Zn^2+^ ions required for MMP activity and inhibited MMPs as shown earlier by others^35,36^, but this needs to be studied in detail.

We also evaluated cellular status in the aorta after adenine diet-induced calcification and after the therapy. Others showed that after adenine diet, SMCs lost phenotype and underwent apoptosis, and those apoptotic bodies aggravated calcification^5,37^. In the *in vivo* studies described here, we saw the loss of SMC phenotype and increased apoptosis in cells close to medial calcification. Surprisingly, we found that after removal of calcification, there was an increase in SMC-type staining in the medial layers of the aorta. Further cell tracing studies are necessary to investigate if the higher SMCs seen after -treatment we due to dedifferentiation or repopulation by recruitment of HSCs, pericytes, or by EMT.

High-frequency ultrasound imaging helped us study changes to the aorta *in vivo*. It was shown that a reduced circumferential strain is an indicator of aortic stiffness^38^. Circumferential strains measured from M-mode images in healthy aortas was as reported in the literature. We observed that circumferential strain is reduced after adenine diet feeding; this indicated increased the aortic stiffness caused by calcification. EDTA NPs treatment removed calcium deposits and improved the circumferential strain levels aortic elasticity, and health.

One important observation was that MMP activity and calcification did not return even after EDTA therapy was suspended for four weeks (EDTA-NP-LT group) although that CKD was present in the animals. We showed earlier that polyphenols such as PGG, when delivered with nanoparticles, do regenerate degraded aortic elastin^39^. Thus, there is an exciting opportunity of dual nanoparticle therapy to first remove calcium deposits using EDTA and then restore medial elastin layers with PGG.

EDTA targeted therapy did not cause any undue side effects in bone and mineral metabolism and biomechanics. Micro CT scanning to study bone morphology showed reduced mineralization in all groups. This was expected, as uremia is associated with reduced bone mineral density^13^. EDTA NPs group showed no additional reduction in mineral density when compared to the Saline-IV and the Blank-NPs group.

In conclusion, we demonstrated that targeted delivery of minimal doses of EDTA is an effective way to reverse calcification in an experimental rat model of CKD. This establishes the enormous potential for targeted EDTA chelation therapy as a viable clinical alternative to reverse vascular calcification in CKD patients.

## Methods

### Preparation of BSA DiR Nanoparticles for *in vivo* targeting studies

DiR (1,1-dioctadecyl-3,3,3,3-tetramethylindotricarbocyanine iodide) (PromoCell GmbH, Heidelberg, Germany) loaded bovine serum albumin (BSA) (Seracare, Milford, MA) nanoparticles were prepared using desolvation method and conjugated to an anti-elastin antibody (US Biological, MA, USA) for targeting purposes as described^39^. Briefly, 250 mg of BSA was dissolved in 4 mL of DI water. 25 mg of DiR dye dissolved in acetone was added to BSA solution and stirred for one hour at room temperature following the addition of glutaraldehyde (EM grade 70%, EMS, PA, USA) at a concentration of 42 µg/mg BSA. The mixture was added dropwise to 24 mL of ethanol while sonicating (Omni Ruptor 400 Ultrasonic Homogenizer, Omni International Inc, Kennesaw, GA) on ice for 30 minutes. Nanoparticles thus obtained were separated by centrifugation and washed. These particles were PEGylated (DSPE-PEG (2000) Maleimide) (Avanti Polar Lipids, Inc., Alabaster, AL) and conjugated to anti-elastin antibody following a previously described protocol^23,24^.

### Preparation of EDTA-loaded nanoparticles for treatment

EDTA-loaded NPs were obtained by dissolving 200 mg of BSA (Seracare, MA) and 100 mg of EDTA (Fisher Scientific, NJ) in 4 mL of deionized water and pH was adjusted to 8.5 with 6 N NaOH. The aqueous solution was added drop-wise to 16 mL of ethanol under probe sonication for 1 hour. For crosslinking, glutaraldehyde at ten µg per mg of BSA was added during sonication. The elastin antibody was conjugated in a method similar to the DiR NPs^23^.

### Animal Studies

#### Experimental renal failure in rats – Adenine rat model

Renal failure was induced in rats by feeding adenine diets having high P and Ca levels. High adenine feeding resulted in crystallization of 2,8-dihydroxyadenine in renal tubules and interstitial spaces, thereby causing nephritis, fibrosis and all metabolic anomalies associated with chronic renal failure^17,18^. Male Sprague-Dawley rats (Charles River Laboratories) were fed standard diets containing 18% protein until their body weights were close to or exceeding 300 g. Animals were then fed a customized adenine (0.75%) diet (Harlan Teklad, Madison, WI, USA) made of 2.5% protein along with higher levels of calcium and phosphorus (1.06% and 0.92% respectively)^16^. Rats were maintained on this diet for 28 days while their body weights and behavior were carefully monitored. If the weight loss was found to be more that 20% of baseline body weight, adenine diet was replaced with rat chow for all the adenine diet-fed animals to maintain consistency and allow them to recover their weights. Adenine diets were reintroduced once the animals recovered their weight, and the total adenine diet feeding time was 28 days. Control rats were fed normal chow diets without adenine for the length of the experiment. Disease progression in live animals was monitored via high-frequency ultrasound imaging (Vevo2100, VisualSonics, Toronto, Canada). Clemson University Animal Research Committee approved all animal use protocols (AUP) for the experimental models. All animals receive humane care in compliance with NIH Public Law 99–158, November 20, 1985, “Animals in Research,” revised in 2015.

### Targeting and Biodistribution of NPs

After 28 days of adenine diet feeding, six rats were injected with elastin antibody conjugated and DiR-loaded NPs via the tail vein. 24 hours later, the rats were euthanized and organs harvested for further analysis. Serum was collected for biochemical analysis using a standard autoanalyzer. The entire body and individual organs were imaged using a Caliper IVIS Lumina XR (Hopkinton, MA) with Ex/Em of 745/795 nm to calculate percentage fluorescence and targeting NPs to the site of injury in the aorta. Percentage Fluorescence was then calculated using the equation:

$$ \% Fluorescence=(\frac{\frac{{fluorescence}\,{in}\,{tissue}}{\,{total}\,{fluorescence}\,{in}\,{all}\,{organs}}\,}{{The}\,{dry}\,{weight}\,{of}\,{tissue}})\ast 100 \% $$

### Treatment with EDTA nanoparticles and intravenous EDTA solution

For the treatment study, rats were fed adenine diets for 28 days and divided into five groups randomly (n = 6 per group). The first group of rats received tail vein injections of PBS to act as a no-treatment control (Saline-IV). The second group of rats received tail vein injections of blank BSA NPs without EDTA, but conjugated with anti-elastin antibody suspended in PBS as controls (Blank-NPs). The third group of rats was given intravenous injections of EDTA solution (EDTA-IV) in PBS twice a week for two weeks at a dosage of 3 mg/kg body weight. The fourth group received tail vein injections of NPs loaded with EDTA and conjugated with anti-elastin antibody suspended in PBS twice a week for two weeks (EDTA-NPs). The last group received EDTA-NPs and was allowed to survive for four weeks after treatment (EDTA-NPs-LT). Animals were euthanized by exsanguination while under isoflurane, aortas (from the arch to the iliac bifurcation) were collected as a unit and fixed in 10% neutral buffered formalin. Other organs including kidneys, hearts, liver, spleen, and lungs were also harvested. Serum was collected from animals of all the groups for biochemical analysis.

### Whole-mount aorta alizarin red S staining

The whole aortic tree from ascending aorta to iliac bifurcation was carefully cleaned of adherent tissues and soaked in freshly made 2% alizarin red solution (pH 4.1–4.3) for 10 minutes and washed with DI water for ten more minutes. The aortas were then imaged by the stereomicroscope (Leica M125 stereo microscope, Leica Microsystems Inc. Buffalo Grove, IL).

### Histology of aorta and kidneys

Aortas and kidneys were fixed in buffered formalin, embedded in paraffin and sectioned per standard procedures. Five-micrometer sections were mounted on positively charged glass slides. Slides were baked overnight at 56 °C in an oven to adhere tissues to the slides and melt the paraffin. Subsequently, the slides were deparaffinized with xylenes followed by dehydration with graded ethanol. Aorta slides were stained with hematoxylin and eosin for overall tissue morphology, Verhoeff-van Gieson (VVG) for elastin fibers and Von Kossa stain with neutral fast red counterstain for calcium deposits. Kidney slides were stained with hematoxylin and eosin for morphology, Periodic acid-Schiff stain (PAS) for polysaccharides and mucosubstances, and with trichrome for muscle and collagen.

### Immunohistochemical analysis of the aorta

Aortas fixed in formalin were embedded in paraffin and sectioned as described earlier. Subsequently, xylenes and graded ethanol were used to deparaffinize the sections, and heat-induced antigen epitope retrieval was performed using citrate buffer (Thermo Scientific, MA). The slides were then incubated overnight at 4 °C with primary antibodies — Mouse anti-Rat Alpha Smooth Muscle Actin (Biolegend, San Diego, CA), Rabbit anti-Rat Caspase-3 (Cell Signaling Technology, Danvers, MA), Rabbit anti-Rat MMP-2 and Rabbit anti-Rat MMP-9 (Enzo Life Sciences, NY). Staining with relevant secondary antibodies was completed using IHC kit (Enzo Life Sciences, NY). Slides were visualized by either 3,3′-Diaminobenzidine (DAB) or 3-Amino-9-ethylcarbazole (AEC) chromogens followed by an appropriate counterstain.

### Quantification of aortic calcium

Calcium content in the aortas was measured after lyophilizing the part of the tissue at renal bifurcation that showed some alizarin red S stain in the whole mount. The lyophilized tissue was hydrolyzed in 6 N HCl at 95 °C and dried under a continuous stream of nitrogen for about 45 minutes. The residue was subsequently reconstituted in 0.01 N HCl and samples were analyzed using the Spectro Acros ICP Spectrometer (SPECTRO Analytical Instruments, Kleve, Germany) at Clemson University Agricultural Service Laboratory.

### Ultrasound analysis of the aorta

A high-frequency ultrasound machine (Vevo2100, VisualSonics, Toronto, Canada) was used to image and monitor the aortas of the rats at different time intervals by utilizing a linear array probe (MS 400D, frequency 18–38 MHz). While being imaged, the animals were given light anesthesia by inhalation of 2% isoflurane and were placed in the supine position on the imaging table. Aortic stiffness parameters circumferential strain and pulse wave velocity (PWV) were calculated from the M-Mode and EKV images obtained with the scans, respectively.

Circumferential Green-LaGrange strain was calculated with the assumption that the strain is uniform around the vessel using the equation given below

$$Circumferential\,Strain=\frac{1}{2}\,({(\frac{Dsystolic}{Ddiastolic})}^{2}-1)\ast 100$$

### Bone integrity as evaluated by micro CT scanning

Femoral bones harvested from the rats were wrapped with parafilm to prevent drying and scanned using a high performance *in vivo* micro CT scanner (Skyscan 1176, Bruker BioSpin, Billerica, MA). Reconstruction was carried out employing a Feldkamp algorithm using the built-in Skyscan Nrecon software. Functional mechanical testing of bone was performed on the rat femurs using a Bose test instrument (Electroforce 3200, Bose, MN, USA) and exposing them to escalating forces until fracture. At fracture, maximum load (N) was determined.

### Statistics

All the results, including graphs in the figures, are given as mean ± S.D. Statistical analysis was performed using a one-way analysis of variance (ANOVA). Results were considered to be significant when p-values ≤ 0.05. Tukey’s HSD was then used post-hoc to identify the treatment groups with a significant difference.

## Supplementary information

Site-specific chelation therapy with EDTA-loaded albumin nanoparticles reverses arterial calcification in a rat model of chronic kidney disease
