# Supplementary material for: Site-specific chelation therapy with EDTA-loaded albumin nanoparticles reverses arterial calcification in a rat model of chronic kidney disease
Source: Sci Rep. 2019 Feb 22;9:2629. doi: 10.1038/s41598-019-39639-8 (PMC6385348; doi:10.1038/s41598-019-39639-8)
Supplement: Supplementary file 1 — Site-specific chelation therapy with EDTA-loaded albumin nanoparticles reverses arterial calcification in a rat model of chronic kidney disease [file 41598_2019_39639_MOESM1_ESM.docx]

**Site-specific chelation therapy with EDTA loaded albumin nanoparticles reverses arterial calcification in a rat model of chronic kidney disease**

**Author names and affiliations:** Saketh R. Karamched^1^, Nasim Nosoudi Ph. D.^2^, Hannah E.Moreland^1^, Aniqa Chowdhury^1^, Naren R. Vyavahare Ph. D.^1*^

**SUPPLEMENTARY DATA**

**
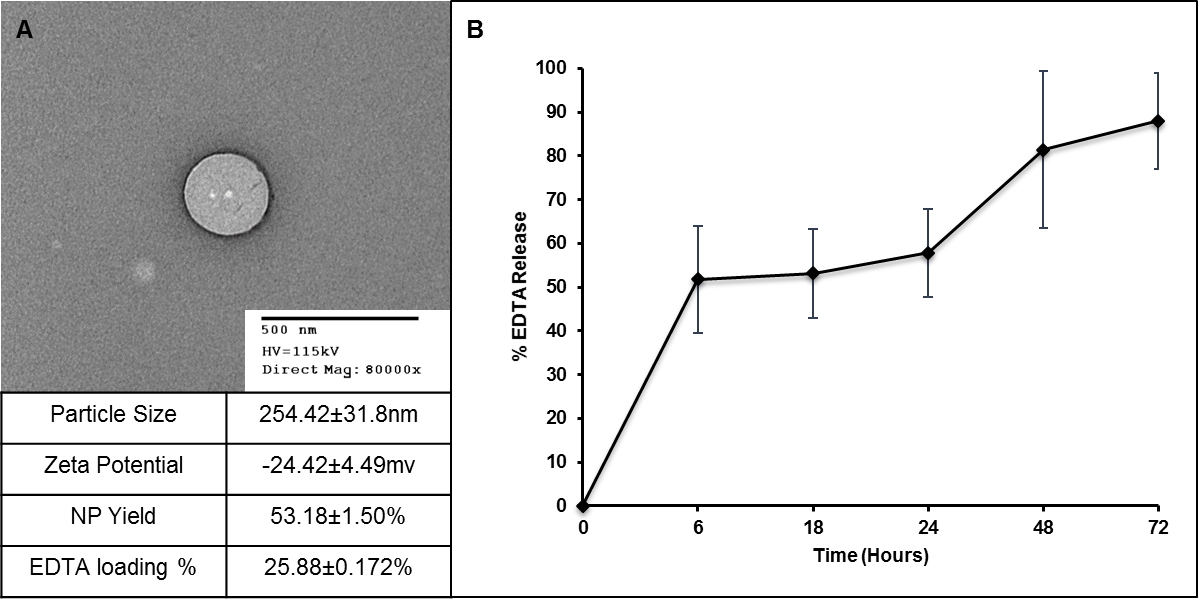
**

**Supplementary Figure S1: EDTA NPs characterization: a.** EDTA NPs characterization. NPs had a final average size of 254.42±31.8nm, which was also confirmed by imaging with TEM. Zeta potential of the NPs was measured as -24.42±4.49mv. The average yield of NPs after centrifugation to purify was 53.18±1.50%. Finally, EDTA loading into the NPs was recorded as 25.88±0.172%

**b**. *In vitro* EDTA release. NPs had a burst release of EDTA with >80% of the drug being released at 72 hours.

**
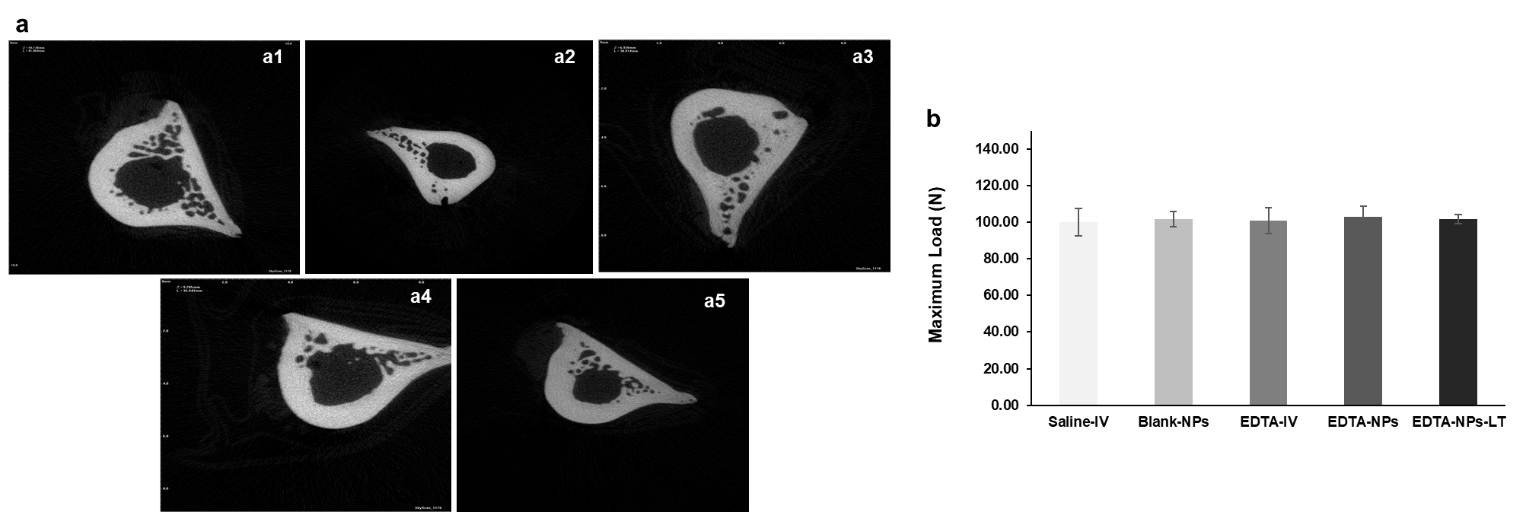
**

**Supplementary Figure S2: Morphology and functional testing of femurs: a**. Bone morphology of femurs from all the treatment groups. Representative two-dimensional slices from microcomputed tomography of rat femurs from the different treatment groups (a1-a4). Bones from all the treatment groups show expected loss of mineralization because of adenine induced uremia. However, there is no additional loss of mineral in the EDTA-IV (a3) and EDTA-NPs (a4) group compared to the Saline-IV (a1) or Blank-NPs (a2) group suggesting that there is no toxic effect of the EDTA treatment at the dosages we have used.

**b**. Maximum load before breaking for femoral bone in the treatment groups. Bones from all the different groups show comparative maximum loads on fracture indicating that there was no adverse effect seen with the EDTA NPs treatment.


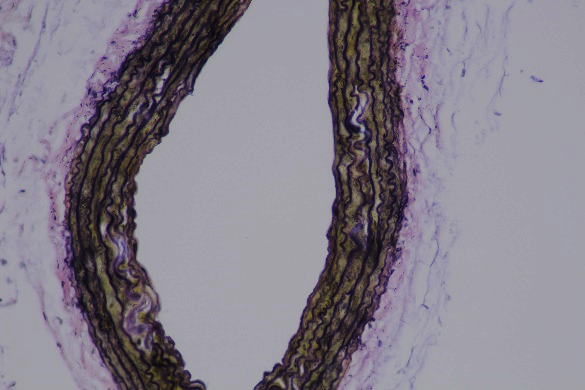

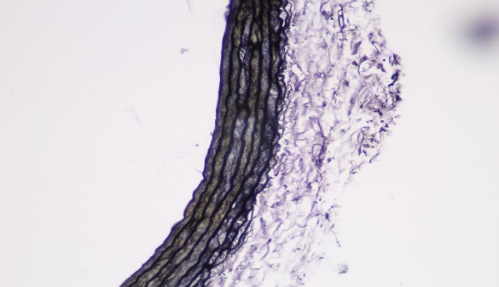

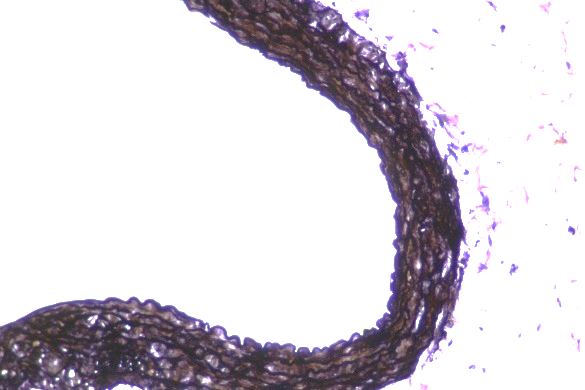


Saline-IV

EDTA-IV

EDTA-NP

**Supplementary Figure S3: VVG stain showing elastin in the aorta after the therapy**

**
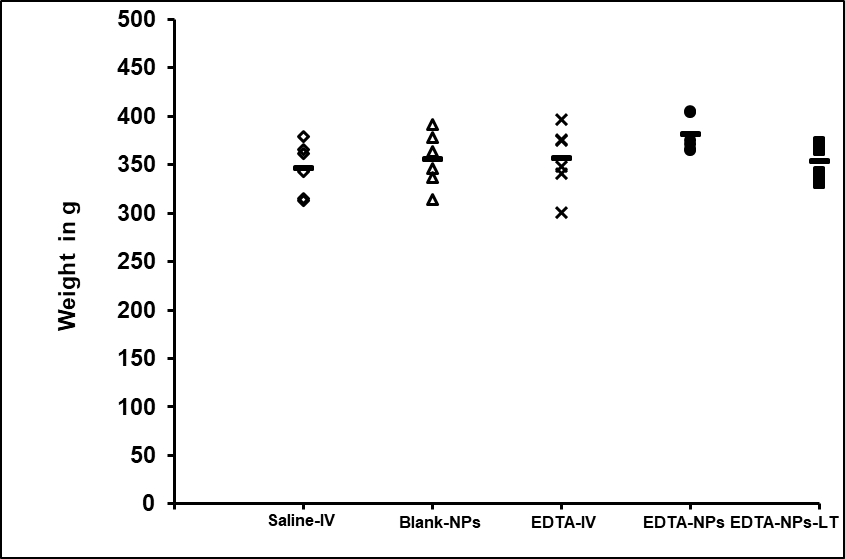


Supplementary Figure S4: Body weights of different groups of adenine diet-fed rats before treatment**
